# Harmonic and power spectrum analysis of vertical ground reaction force in patients who have a stroke within 3 months

**DOI:** 10.1113/EP093186

**Published:** 2026-03-13

**Authors:** Li Zhang, Hongfang Yao, Shuisheng Fu, Jung Hung Chien

**Affiliations:** ^1^ Department of Rehabilitation Medicine The First Affiliated Hospital of Guangxi Medical University Nanning Guangxi China; ^2^ Dr. Sid E. Williams Center for Chiropractic Research Life University Marietta Georgia USA

**Keywords:** Fourier analysis, power spectrum analysis, stroke survivors

## Abstract

Stroke remains a primary cause of disability globally. An in‐depth comprehension of gait impairments following a stroke is vital for crafting effective therapeutic interventions, and vertical ground reaction forces (vGRF) provide valuable insights into these mechanics. Eighteen stroke survivors within 90 days post‐stroke and 18 healthy controls participated in this study, completing a 2‐min walking trial on an instrumented treadmill to investigate vGRF signals using harmonic and power spectrum analysis. Significant interactions were observed in vGRF patterns between stroke survivors and controls, and between paretic and intact legs. The paretic leg of stroke survivors exhibited a significantly lower harmonic coefficient $A_{0}$ (paretic: 61.71 ± 4.09% body weight vs. intact: 74.77 ± 6.65% body weight), a lower essential number of harmonics (paretic: 7.57 ± 1.12 vs. intact: 10.87 ± 1.76), a lower 99.5% power frequency (paretic: 4.19 ± 0.10 Hz vs. intact: 4.71 ± 0.31 Hz), and a higher median power frequency (paretic: 0.43 ± 0.03 Hz vs. intact: 0.40 ± 0.01 Hz) compared to the intact leg. The paretic leg demonstrated a simplified waveform shape (inverse U) compared to the intact leg (M shape), which likely contributes to the reduced essential number of harmonics and 99.5% power frequency, and the paradoxical increase in median frequency. These findings quantify the mechanical consequences of post‐stroke neuromuscular deficits and highlight the potential of frequency domain analysis as a diagnostic tool, offering important implications for developing personalized rehabilitation strategies to improve outcomes in subacute stroke survivors.

## Abstract (highlights)

- **What is the central question of this study?**
  How do the harmonic and power spectrum characteristics of vertical ground reaction force (vGRF) differ between patients who have experienced a stroke within the past 3 months and healthy controls during walking?
- **What are the main findings and its importance?**
  Stroke survivors showed altered frequency components and decreased harmonic complexity in their vGRF patterns, indicating less stable and less rhythmic walking mechanics. Unlike standard time‐based gait measures, these frequency‐domain features can detect more subtle, early or persistent abnormalities in neuromuscular control that might not be apparent on simple observation.

## 1 INTRODUCTION

Stroke remains a critical global health concern, exacting a substantial toll on individuals, healthcare systems and societies worldwide. Recent data from 2019 reveal approximately 12.2 million new stroke cases globally, resulting in 143 million disability‐adjusted life years lost and 6.6 million deaths (eClinicalMedicine, 2023). This high prevalence and patient burden translate into substantial financial costs, encompassing expenses for treatment, rehabilitation, social care and lost productivity. Initiating rehabilitation within 24–48 h post‐stroke has been shown to significantly reduce complications such as deep vein thrombosis, pressure sores and muscle contractures (Li et al., 2024). Moreover, early rehabilitation is associated with improved functional outcomes and lower mortality rates in the first 90 days (Li et al., 2024). However, standard clinical assessments may not fully capture subtle deficits in dynamic stability and gait smoothness without the use of motion capture systems (Hulleck et al., 2022), which are typically unaffordable for small clinics due to budget and space constraints. Alternatively, analysing the frequency content of ground reaction forces (GRF), a fundamental variable in biomechanics, using less expensive force plates or an instrumented treadmill offers a sensitive and accessible metric to detect these underlying impairments in balance control during walking. Given the substantial impact of stroke and the potential benefits of early intervention, there is a pressing need for cross‐sectional studies to establish foundational knowledge about the biomechanical differences between stroke survivors in the early stage and healthy controls.

Vertical ground reaction force (vGRF) is the predominant component of ground‐generated forces during gait. Characteristically, healthy gait exhibits an M‐shaped bimodal profile where the two maxima, corresponding to weight acceptance (following heel strike) and push‐off (preceding toe‐off), typically reach approximately 1.2 body weights (BW), while the intervening mid‐stance valley decreases to a minimum of approximately 0.8–0.9 BW (Jiang et al., 2020; Martínez‐Pascual et al., 2023; Yu et al., 2021). Traditionally, studies involving chronic stroke survivors have relied on discrete variables, specifically the magnitude and timing of these peaks, to quantify gait differences (Kokolevich et al., 2021). On the other hand, survivors of subacute stroke often show an abnormal characteristic. Instead of the normal M‐shape, subacute stroke patients tend to have a distorted inverse U shape vGRF profile during the stance phase of the paretic leg (Chen et al., 2007). This can be accounted for by impaired neural control and muscle weakness. This altered profile reflects underlying deficits in neural control and muscle weakness. This deviation is critical because the vGRF profile serves as a direct indicator of motor capacity. Observable changes in the waveform, such as a flattened active peak, allow researchers to infer specific pathologies like propulsive failure or instability during the weight acceptance phase. Quantitative analysis of these subjective observations is currently not possible using conventional peak approaches. As the two peaks coalesce to form one peak or hump in individuals that have suffered an acute stroke, conventional peak analysis is no longer valid. There is clearly a need for further analysis.

A primary advantage of harmonic decomposition is its ability to reveal gait pathologies that conventional peak measures often miss (Schneider & Chao, 1983; White et al., 2005; Wu et al., 2014). Instead of simply tracking how force magnitude changes over the gait cycle, this technique uses Fourier analysis to break the vGRF profiles down into its component frequencies. This uncovers critical details about gait rhythm, smoothness and energetic cost, nuances that are frequently invisible to traditional observation or discrete variable calculations. This capability is particularly valuable for quantifying the shift from a healthy ‘M‐shaped’ profile to the pathological ‘inverse U’ or single‐hump pattern often seen during stroke recovery. The clinical relevance of this spectral analysis is well‐established. Research shows that individuals with spinal cord injuries exhibit a disproportionately high first harmonic component compared to healthy controls, who typically rely more on the second harmonic component (Fineberg et al., 2013). This spectral shift implies that these patients are operating with gross motor patterns at fundamental frequencies, lacking the fine‐tuned control required for physiologic walking. Similar frequency distortions have been mapped in children with cerebral palsy (White et al., 2005) and patients with knee pathology (Schneider & Chao, 1983).

Building on the established potential of harmonic decomposition to quantify gait deficits, this study seeks to extend these analytical methods to a specific and critical population: stroke survivors within the first 90 days of recovery. Specifically, this investigation utilizes harmonic and frequency analysis of the vGRF to address three primary objectives: (1) to mathematically characterize the differences in vGRF profiles between healthy controls and subacute stroke survivors (≤3 months post‐stroke) by determining the number of Fourier coefficients required for accurate reconstruction in this specific population; (2) to examine the variability of vGRF characteristics using these spectral metrics; and (3) to evaluate the clinical utility of harmonic analysis as a tool for distinguishing gait impairments in the early post‐stroke phase.

## 2 METHODS

### 2.1 Ethical approval

This study was conducted in strict adherence to the principles outlined in the *Declaration of Helsinki*. Ethical approval was granted by the Medical Ethics Committee of the First Affiliated Hospital of Guangxi Medical University (Approval No. KY‐E‐271). Furthermore, the reporting of this study complies with the Strengthening the Reporting of Observational Studies in Epidemiology (STROBE) guidelines for cross‐sectional studies. This study was not registered in a public clinical trial database as it was a cross‐sectional observational study.

### 2.2 Participants

**FIGURE 1:**
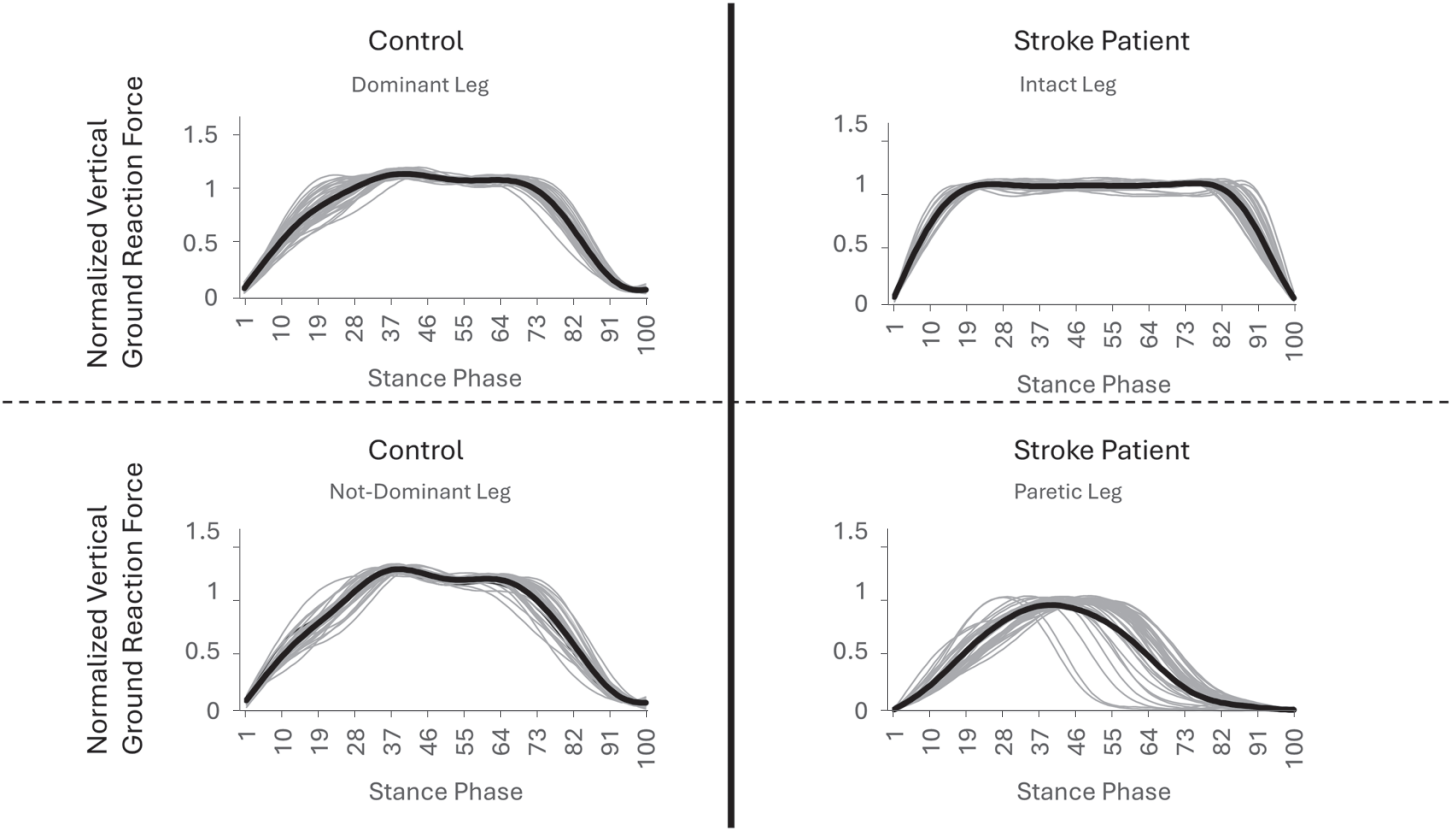
The shape of vertical ground reaction force in a stroke survivor and a matched control in intact/paretic or dominant/not‐dominant leg.

Eighteen patients with stroke who had a stroke within 3 months and 18 healthy controls were recruited in this study (patients: age: 62.9 ± 14.3 years, height: 170.5 ± 7.2 cm, weight: 71.8 ± 11.6 kg, walking speed: 0.85 ± 0.51 km/h, time from stroke to data collection: 50.9 ± 19.5 days, 15 males/3 females; controls: age: 63.2 ± 14.3 years, height: 170.0 ± 6.7 cm, weight: 71.7 ± 11.2 kg, walking speed: 2.26 ± 0.77 km/h, 15 males/3 females). More details are shown in Figure 1. Inclusion criteria were for patients: (1) less than 3 months post‐stroke, (2) a single and unilateral hemiplegia, (3) ability to walk a minimum of 10 m independently, and (4) a score of Functional Independence Measure Locomotor Item ≥5. Moreover, to be eligible for participation, stroke survivors were required to achieve a minimum score of 16 out of 34 on the lower extremity Fugl–Meyer Assessment (LE‐FMA). This inclusion threshold was adopted based on criteria established by Malone & Bastian (2014), who demonstrated that this level of motor function is sufficient to tolerate treadmill walking protocols. For controls: (1) no orthopaedic impairment, which causes unstable gait or chronic pain in the musculoskeletal system affecting the gait, and (2) no cognitive disorders. Exclusion criteria included (1) evidence of moderate to severe chronic white matter disease on MRI, (2) lower extremity joint replacement, (3) any pain that limited walking ability, (4) cognitive disorders, (5) dizziness, and (6) orthopaedic conditions that impaired walking ability. The sample size for this study was determined based on previous research utilizing similar experimental paradigms in stroke survivors (Regnaux et al., 2008; Zhang et al., 2022). First, Regnaux et al. (2008) demonstrated that a sample of 10 participants was sufficient to observe significant training effects, specifically improvements in walking velocity, in stroke survivors 3–6 months post‐stroke. Although the authors acknowledged the small sample size as a potential limitation, a *post hoc* power analysis validated the sufficiency of their cohort; a difference in walking velocity of 0.036 m/s was detected with 98% statistical power (Type I error α = 0.05). This confirms that a sample of 10 was adequate to detect meaningful gait performance changes. Secondly, Zhang et al. (2022) substantiated the sufficiency of a sample size of 15 per group through the analysis of effect sizes using the partial eta squared ($\eta_{p}^{2}$) method. Their data revealed substantial effect sizes for key kinematic variables in the paretic leg, yielding $\eta_{p}^{2}$ values of 0.314 for maximum hip extension and 0.203 for maximum hip flexion. According to Cohen's guidelines, where 0.138 represents a large effect, these results were interpreted as moderate‐to‐large. Consequently, a cohort of 15 stroke survivors and 15 healthy controls was deemed sufficient to statistically detect significant biomechanical alterations during treadmill walking. Therefore, the recruitment of 18 stroke survivors and 18 controls in the current study provides a robust sample size to investigate changes in the harmonics of vGRF.

### 2.3 Experimental equipment

vGRF data were collected using a pressure‐sensing treadmill (Zebris FDM‐T, Zebris Medical GmbH, Isny, Germany). The treadmill features a 150 × 50 cm running surface equipped with a high‐density matrix of 10,240 calibrated capacitive sensors. While the system primarily measures plantar pressure distribution, vGRF was derived by integrating the pressure values over the contact area of the active sensors (*F* = *P* × *A*). Data were recorded at a sampling frequency of 100 Hz and processed using the integrated Zebris FDM‐T Analysis software. This method of deriving vGRF from high‐resolution pressure data has been previously established in clinical research. For instance, the Zebris FDM‐T has been employed to analyse force‐related parameters in patients with multiple sclerosis (Kalron et al., 2013) and to compare vGRF profiles in preadolescents with Down's syndrome (Wu & Ajisafe, 2014). Crucially for the present study, this specific instrumentation was previously validated for the extraction of harmonic numbers, Fourier coefficients and power spectrum frequency characteristics (Wu et al., 2014), supporting its validity for the frequency‐domain analyses conducted here.

### 2.4 Experimental protocol

Upon signing the informed consent form, participants were assigned a treadmill practice session to familiarize themselves with walking on the treadmill. During this familiarization, a walking‐on‐treadmill trial was used to determine each participant's preferred walking speed. The process involved several steps: participants initially stood beside the treadmill, holding the handrail, and then stepped onto the belt as it accelerated to 0.5 km/h. Once confident, they were encouraged to walk without holding the handrail. Participants were asked if the current speed felt similar to their regular and comfortable pace, and if necessary, the speed was adjusted by increments of 0.1 km/h until they agreed it was comfortable. This process was repeated until participants confirmed their preferred speed, after which they continued walking at this speed for 1 min to finalize the familiarization process. Following familiarization, participants were given a mandatory 5‐min rest. Next, they completed three 2‐min walking trials on the treadmill (Kempski et al., 2018).

### 2.5 Harmonic and power spectrum analysis of vGRF

**FIGURE 2:**
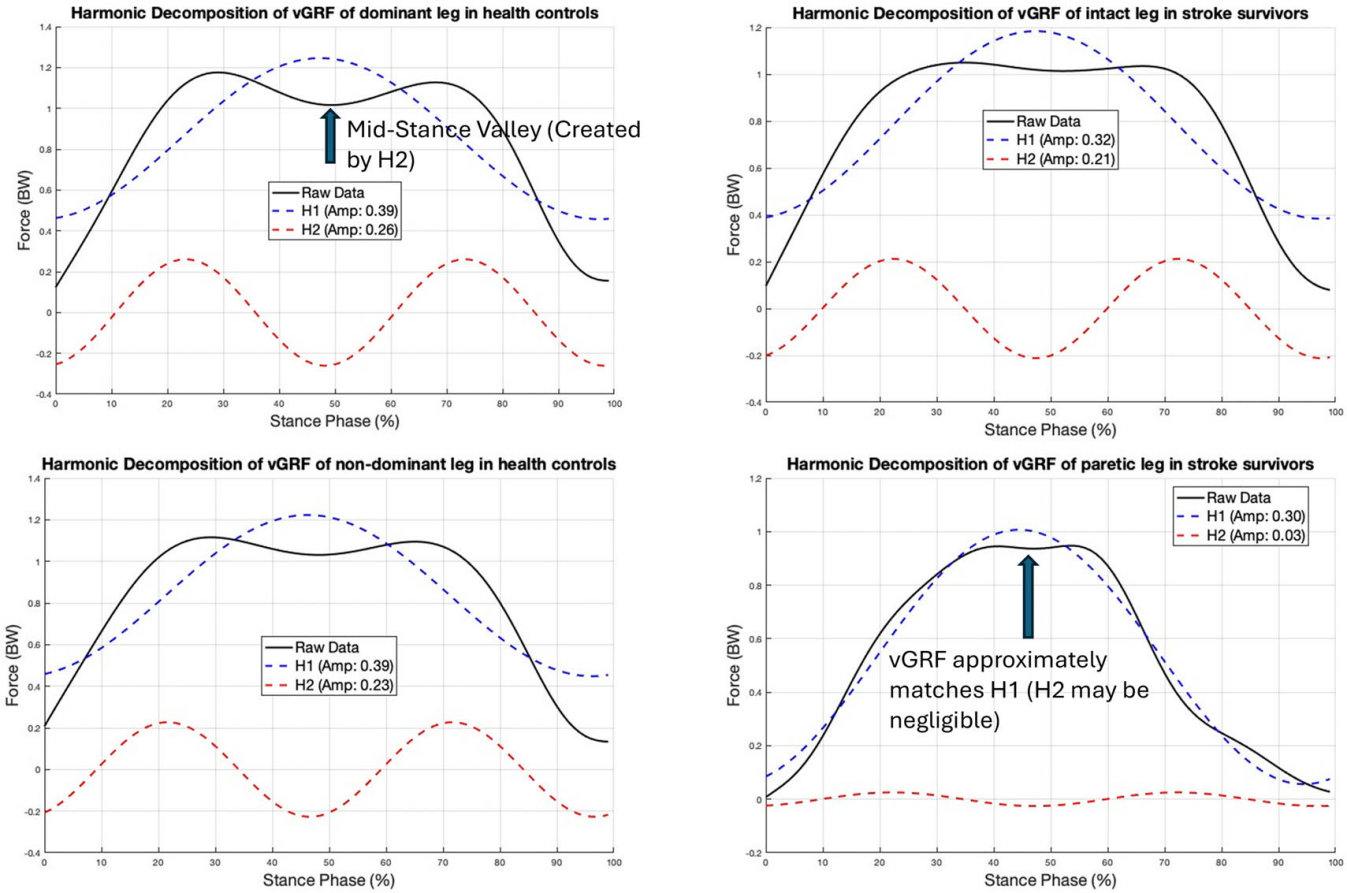
Harmonic decomposition workflow of the vertical ground reaction force (vGRF). The raw vGRF signals (black continuous lines) were decomposed into their primary sinusoidal components: the first harmonic (*H*_1_, blue dashed lines) representing the fundamental weight‐bearing arch, and the second harmonic (*H*_2_, red dashed lines) representing the dynamic unloading force. Left panels, healthy controls: the characteristic bimodal ‘M‐shape’ is achieved through the constructive interference of *H*_1_ and a significant component *H*_2_ (amplitude ≈ 0.23–0.26 body weight (BW)), which creates the necessary mid‐stance valley. Bottom right panel, stroke paretic leg: the pathological ‘inverse U’ profile is dominated almost exclusively by *H*_1_ (amplitude: 0.30 BW), while the component *H*_2_ is negligible (amplitude: 0.03 BW). This spectral simplification confirms that the paretic limb acts as a primitive pendulum (fundamental frequency only) lacking the higher‐frequency modulation required for dynamic weight acceptance.

The basis of Fourier series was that a complex periodic waveform (vGRF–time curves) can be analysed into a number of harmonically related sinusoidal waveforms. In the analysis of each vGRF signals with *N* sample points during the period *T*, a vGRF can be expressed with a finite number of harmonics in the series, termed as discrete Fourier transform (DFT):

$$F\;\left(t\right)=A_{0}+\sum_{n=1}^{N/2}\left(A_{n}\cos\frac{2\pi nt}{T}+B_{n}\sin\frac{2\pi nt}{T}\right)$$

$$\;A_{0}=\frac{1}{N}\sum_{N=0}^{N/2}F\left(t\right)$$

where *n* is the harmonic number, *t* is time (s), each of the frequency domains of vGRF is *N*/2 + 1 long, and the maximum number required to reconstruct the vGRF is given by Nyquist criterion is (*N* − 1)/2 (Hamill et al., 1997). The parameter $A_{0}$ (percentage of BW), represents the mean value of the vGRF, signal calculated exclusively over the stance phase, rather than the entire gait cycle. This distinction is critical for interpretation: had it been calculated over the complete gait cycle (including the swing phase where force is zero), its value would theoretically equal BW (100% of BW) to maintain vertical equilibrium. However, in the present study, it specifically quantifies the average magnitude of the vertical force sustained during the period of ground contact (stance phase, White et al., 2005). Thus, the amplitude could be smaller than BW (100% of BW), depending on whether the gait was pathological or normal (White et al., 2005). For biomechanical reasons, while the mean vertical force averaged over the entire gait cycle must equal 100% BW to maintain dynamic equilibrium, *A*_0_ represents the mean force calculated exclusively over the stance phase in the present study. Because the gait cycle includes a double‐support phase where BW is distributed between both limbs, the mean force on a single leg during its stance period is characteristically less than BW. Additionally, special focus was placed on the first two harmonics of vGRF, which primarily dictate the shape of the force curve. Additionally, special focus was placed on the first two harmonics of vGRF, which primarily dictate the shape of the force curve. The first harmonic (*H*_1_), representing the fundamental frequency of the stance phase, corresponds to the primary weight‐bearing arch. It creates a simple, unimodal (inverse U) trajectory with a single peak near the mid‐stance. The second harmonic (*H*_2_), oscillating at twice the fundamental frequency, is biomechanically responsible for the dynamic unloading during mid‐stance. In a healthy gait pattern, the superposition of a significant component onto the arch creates the characteristic ‘valley’ between the heel‐strike and push‐off peaks (the bimodal ‘M‐shape’). Consequently, the relative dominance of these components determines the complexity of the gait profile. To quantify this, we calculated the harmonic amplitude ratio (*H*_1_/*H*_2_). This ratio serves as a shape index: a lower ratio reflects a healthy bimodal profile (where it is significant enough to create a valley), while a significantly elevated ratio indicates the suppression of the second harmonic, mathematically characterizing the pathological shift toward a smoothed, unimodal (inverse U) profile (Figure 2 ). Additionally, based on the study by Schneider & Chao (1983), the essential number of harmonics (*n*_95_) was defined as what satisfied the condition where the sum of the relative amplitude of each harmonic over the total amplitude was ≤0.95:

$$\sum_{n=1}^{n_{95}}\frac{\sqrt{A_{n}^{2}+B_{n}^{2}}}{\sum_{n=1}^{m}\sqrt{A_{n}^{2}+B_{n}^{2}}}\;\leq 0.95$$

**FIGURE 3:**
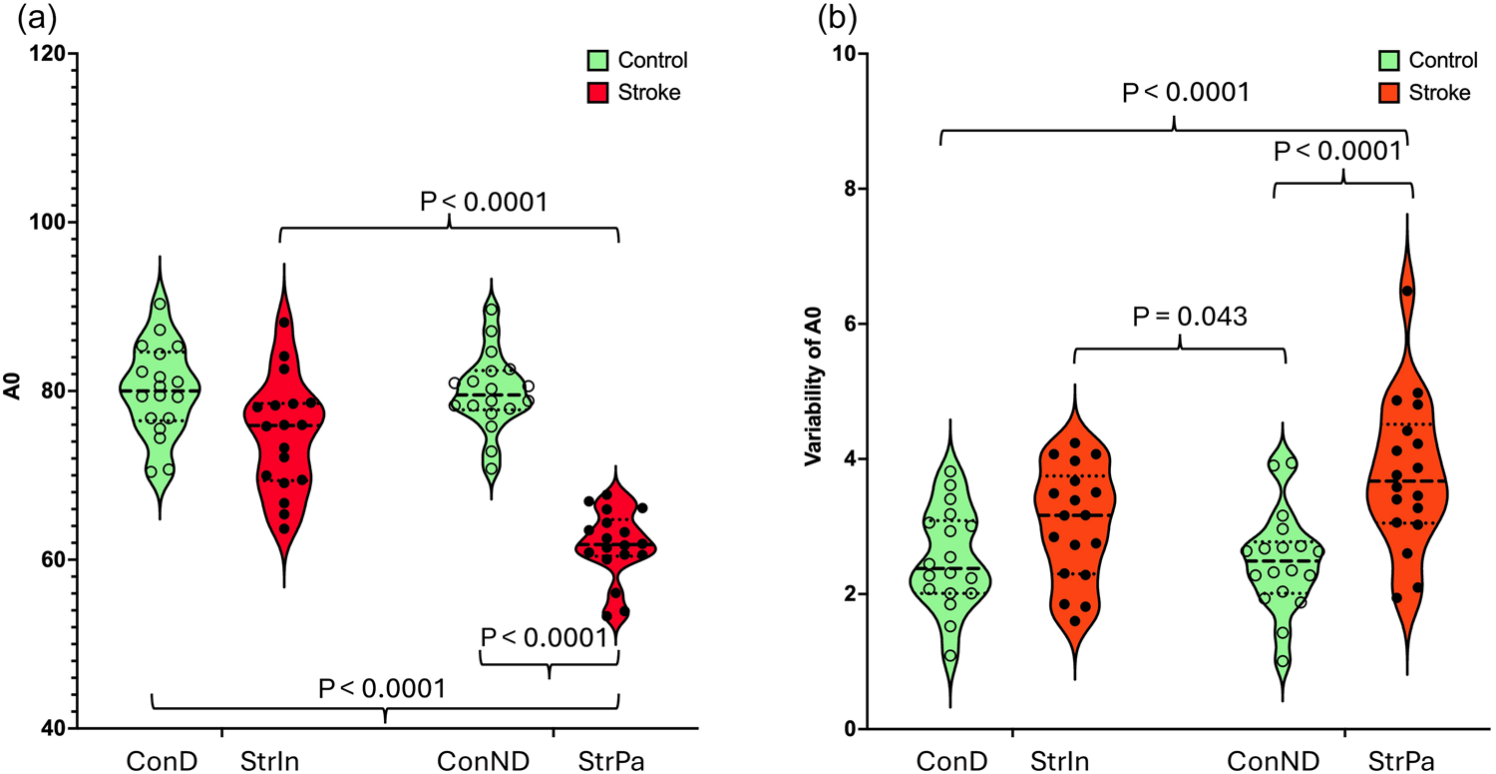
The harmonic coefficient *A*_0_ (percentage body weight). (a) The mean magnitude of *A*_0_ representing the area under the force‐time curve during stance. (b) The variability (standard deviation) of *A*_0_ across steps. ConD, dominant leg of controls; ConND, non‐dominant leg of controls; StrIn, intact leg of stroke survivors; StrPa, paretic leg of stroke survivors.

**FIGURE 4:**
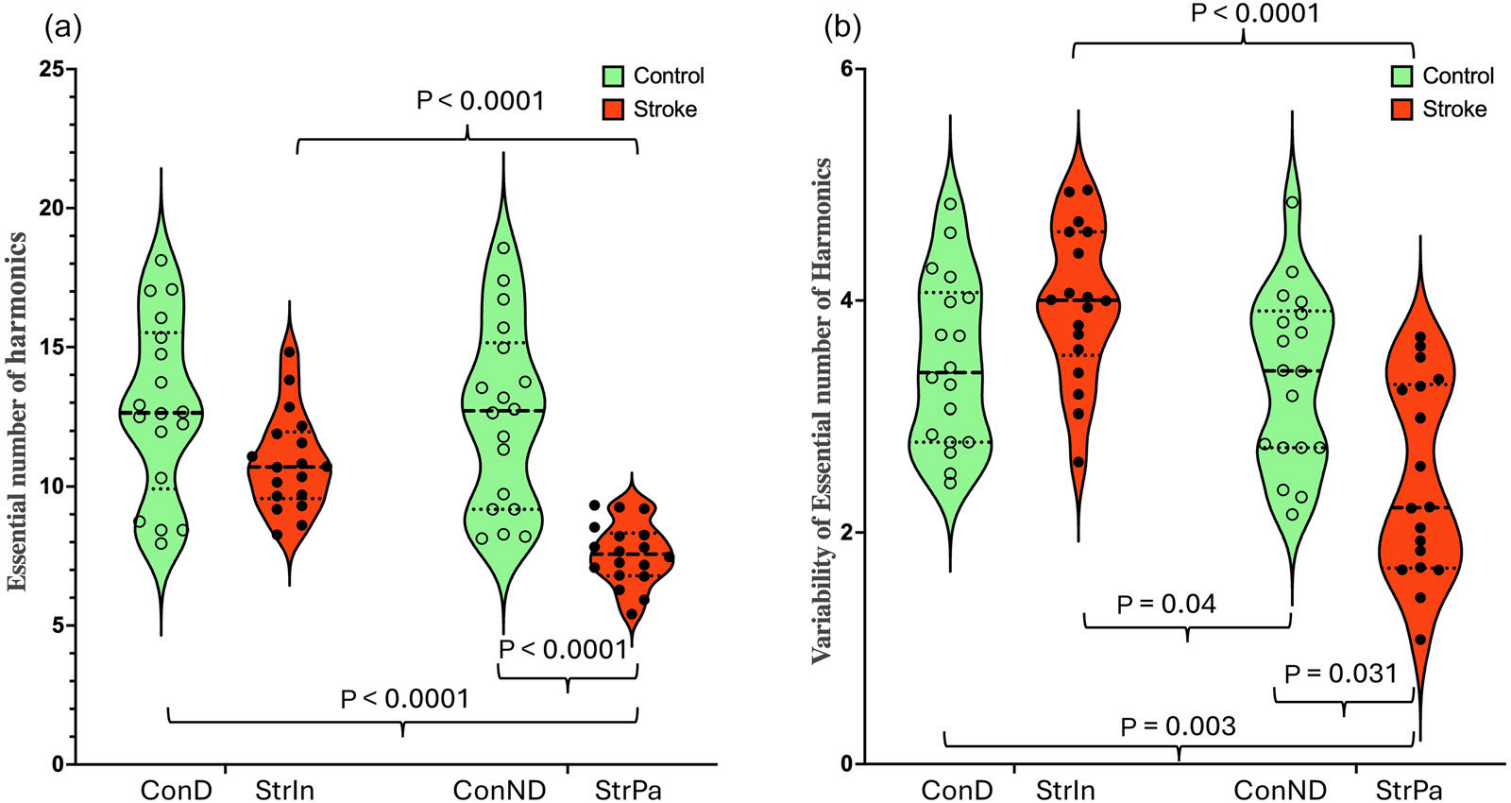
The essential number of harmonics. (a) The mean number of harmonics required to reconstruct 95% of the original signal power. (b) The variability of the essential number of harmonics. ConD, dominant leg of controls; ConND, non‐dominant leg of controls; StrIn, intact leg of stroke survivors; StrPa, paretic leg of stroke survivors.

**FIGURE 5:**
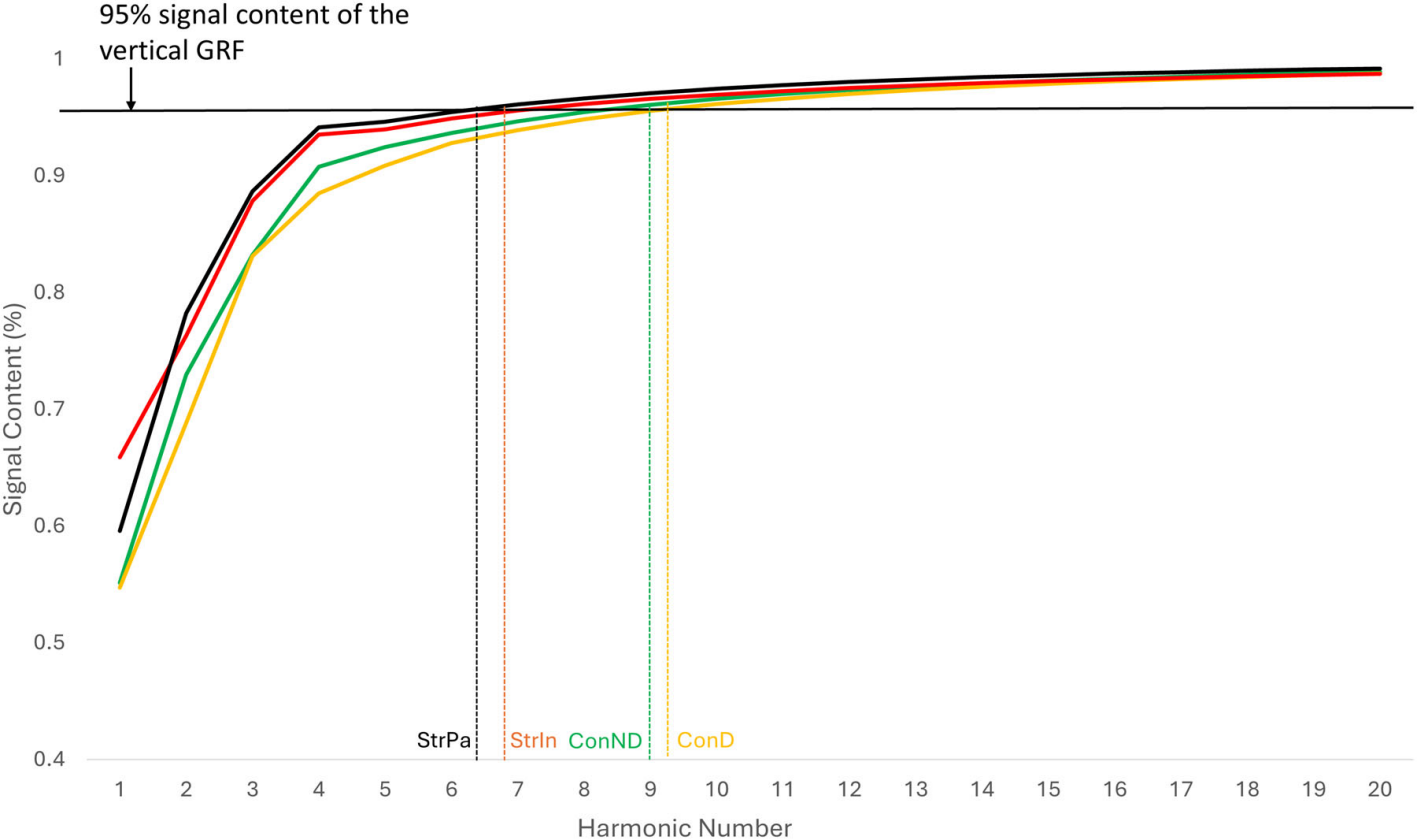
An essential number of harmonics required to reconstruct the original force–time signal for a stroke survivor and a matched control. ConD, dominant leg of controls; ConND, not‐dominant leg of controls; GRF, ground reaction force; StrIn: intact leg of stroke survivors; StrPa, paretic leg of stroke survivors.

**FIGURE 6:**
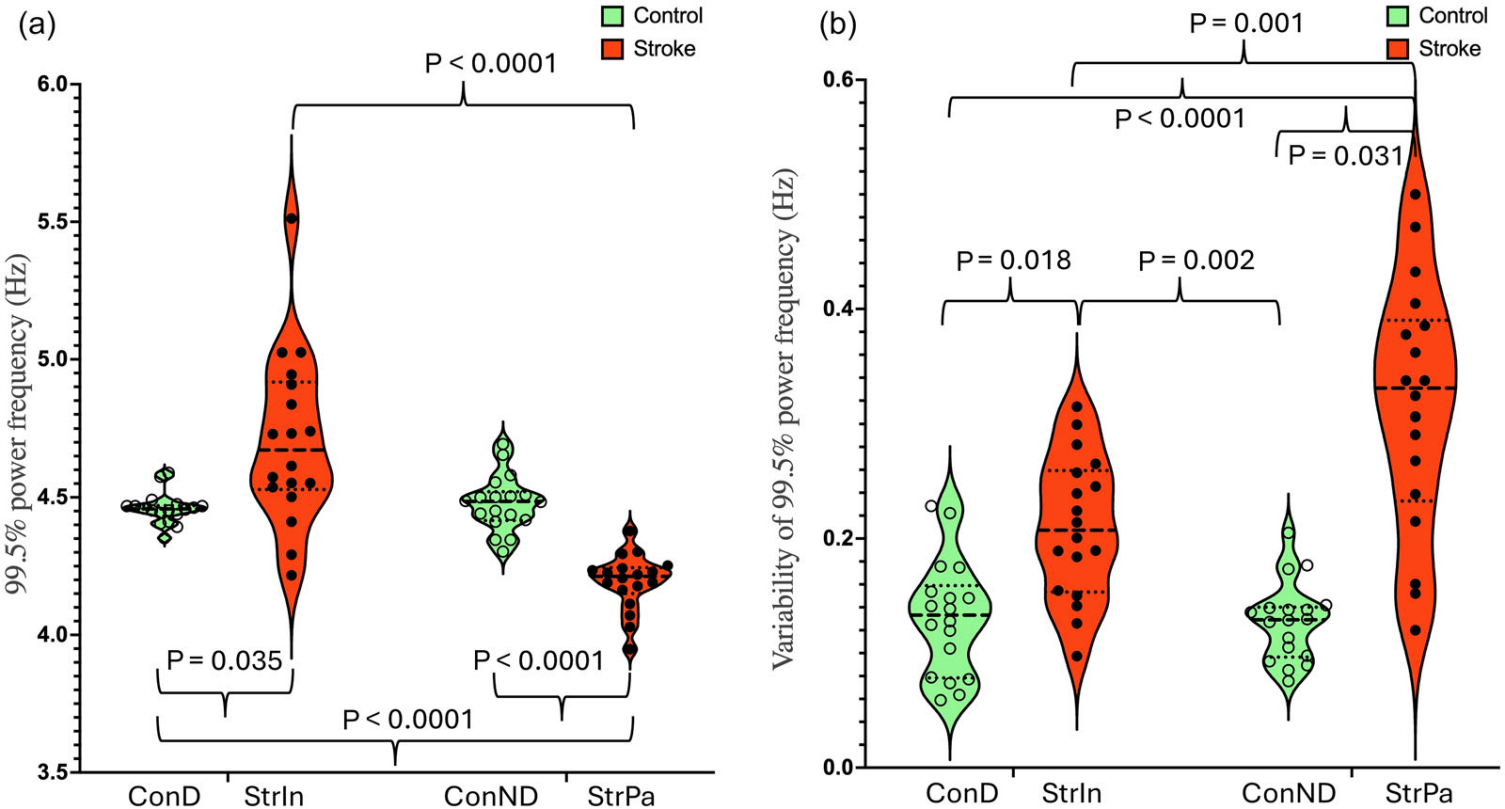
The 99.5% power frequency. (a) The mean frequency bandwidth containing 99.5% of the signal power. (b) The variability of 99.5% power frequency. ConD, dominant leg of controls; ConND, non‐dominant leg of controls; StrIn, intact leg of stroke survivors; StrPa, paretic leg of stroke survivors.

**FIGURE 7:**
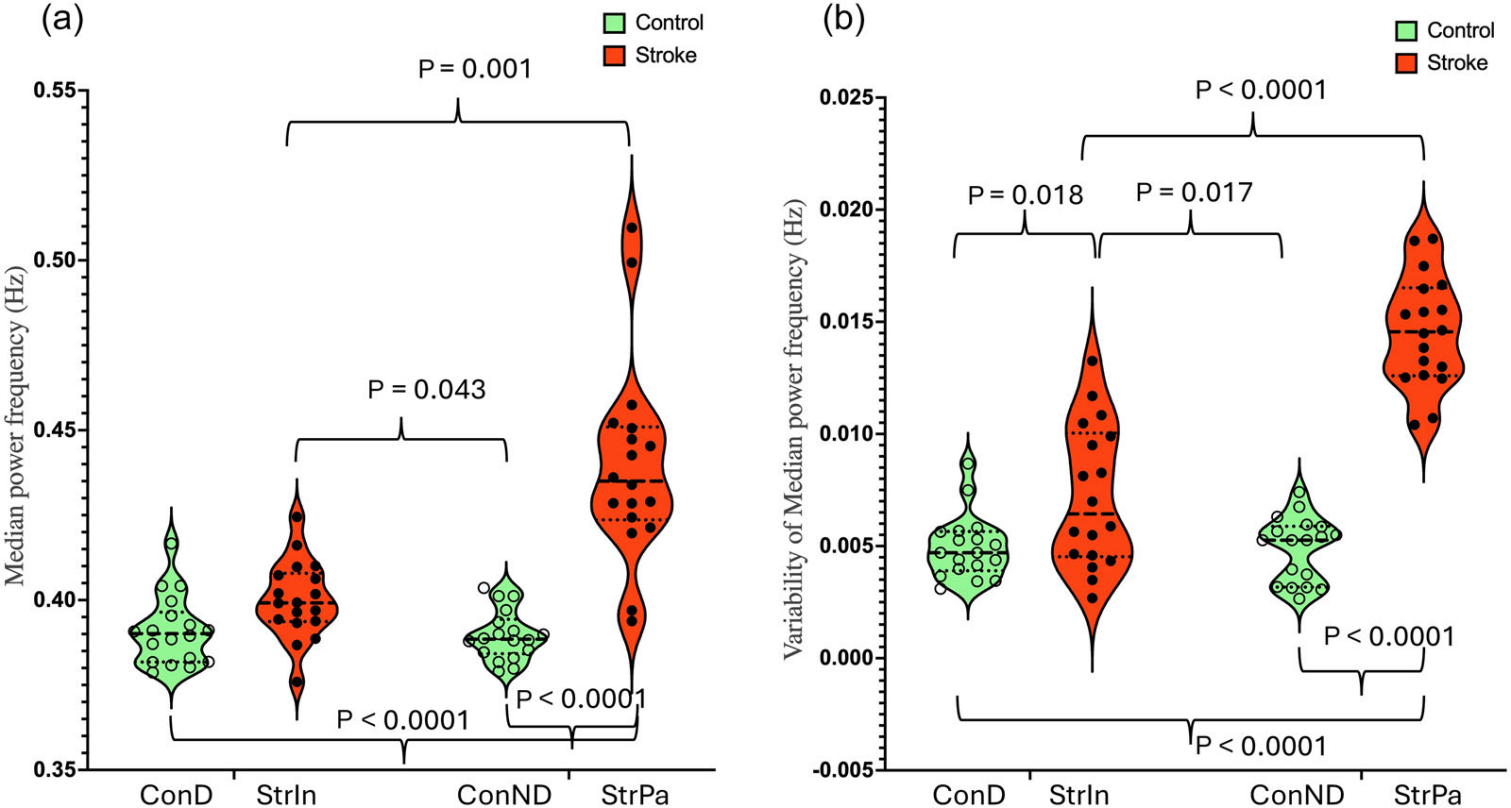
The median power frequency. (a) The mean frequency dividing the power spectrum in half. (b) The variability of the median power frequency. ConD, dominant leg of controls; ConND, non‐dominant leg of controls; StrIn, intact leg of stroke survivors; StrPa, paretic leg of stroke survivors.

From the power spectrum, the median power frequency and 99.5% power frequency were applied to investigate the vGRF between groups and within different legs in preadolescents with Down's syndrome (Wu et al., 2014), in patients with multiple sclerosis (Wurdeman et al., 2011) and in patients with peripheral arterial disease (McGrath et al., 2012).

The 99.5% power frequency (*f*_99.5_) was that which contained 99.5% of vGRF as follows:

$$\int_{0}^{f_{99.5}}P\left(f\right)df=0.995\int_{0}^{f_{\max}}P\left(f\right)df$$

where *P* is the power calculated as the integral of the frequency and amplitude of vGRF, and *f*_max_ is the maximum frequency of the signal.

Median power frequency (*f*_median_) is the point where half of the total power was above and below that frequency as follows:

$$\int_{0}^{f_{\mathrm{median}}}P\left(f\right)df=\int_{f_{\mathrm{median}}}^{f_{\max}}P\left(f\right)df$$

### 2.6 Data reduction

vGRF data were acquired using the Zebris FDM‐T pressure‐sensing treadmill system (Zebris Medical GmbH), operated via the integrated Zebris FDM software suite. Measurements were recorded at a sampling frequency of 100 Hz using the ‘Gait Analysis’ module. Prior to each session, a zero‐measurement was performed on the unloaded treadmill to calibrate the force sensors. To ensure the analysis of steady‐state gait, the recording interval was refined in the software's ‘View’ mode; the initial acceleration and final deceleration phases were excluded by adjusting the time–force diagram boundaries, retaining only the stable walking phase for analysis. Step segmentation was verified visually, and the ‘Manual Step Definition’ function was utilized to correct any misidentified foot contacts. For the purpose of harmonic decomposition, raw vGRF data were extracted using the ‘XML (raw data)’ export interface. Unlike standard clinical reports that provide discrete variables, this export format generated continuous force–time series data for every measured time point, preserving the temporal resolution required for subsequent Fourier analysis. Notably, previous studies utilizing harmonic analysis (Schneider & Chao, 1983; White et al., 2005; Wu et al., 2014) and frequency domain analysis (McGrath et al., 2012; Wurdeman et al., 2011) did not report whether a filter was applied to the vGRF data. To address this, prior to harmonic decomposition, the raw vGRF time series data were filtered using a fourth‐order, zero‐lag, low‐pass Butterworth filter with a cutoff frequency of 8 Hz (Masani K et al., 2002; Winter, 1990). This filtering threshold was selected to attenuate high‐frequency mechanical artifact and sensor noise while preserving the physiological frequency content essential for characterizing the pathological ‘inverse U’ waveform. Subsequently, the filtered vGRF data were processed through harmonic and power spectrum analysis using custom‐written code in MATLAB (MathWorks, Natick, MA, USA). To ensure consistency across the cohort and minimize the potential for fatigue‐induced modifications to the vGRF patterns, the analysis was standardized to 40 stance phases per leg (80 total steps). This threshold was determined based on the minimum number of completed cycles observed within the stroke group. Variability was defined as the standard deviation calculated across these 40 stance phases for each leg. Stance phase was defined as the interval from heel strike to toe‐off. Heel strike was identified as the first time point where the filtered vGRF signal deviated from the zero baseline, and toe‐off as the return to zero (Wu & Ajisafe, 2014; Wu et al., 2014). Unlike strain‐gauge force platforms that often require a higher threshold (e.g., 10 N) to account for signal drift and baseline noise, the capacitive sensor matrix of the FDM‐T system demonstrated a distinct, stable zero baseline between stance phases. Consequently, a 0 N threshold was deemed appropriate to maximize temporal accuracy. To prevent the inclusion of artifacts, all identified gait cycles were visually verified using the software's playback function. The dominant leg was defined as the preferred leg to kick a ball.

### 2.7 Statistical analysis

An independent Student's *t*‐test was used to compare preferred walking speed, height, weight and age between stroke survivors and healthy controls. The Shapiro–Wilk test assessed normal distribution of the data. For normally distributed data, a mixed two‐way repeated measures ANOVA was employed to examine the interaction between the effects of stroke and different legs on dependent variables as follows: $A_{0}$, variability of $A_{0}$, essential number of harmonics, variability of essential number of harmonics, 99.5% power frequency, variability of 99.5% power frequency, median power frequency, variability of median power frequency, and *H*_1_/*H*_2_ ratio. If the data were not normally distributed, Friedman's test was used instead. In cases of significant interaction, pairwise comparisons were conducted using the Tukey method for normally distributed data or the Mann–Whitney test for between‐group comparisons and the Wilcoxon test for within‐group comparisons across different inclines for non‐parametric data. The α‐level was set at 0.05. The effect size was defined by partial eta squared values (>0.14: large effect size, 0.06: medium effect size, and 0.01: small effect size).

## 3 RESULTS

### 3.1 Participants’ information

The only significant difference between stroke survivors who had a stroke within 90 days and healthy controls was the preferred walking speed (0.85 km/h vs. 2.26 km/h, *P* < 0.001). More details are shown in Table 1.

**TABLE 1:** Participants’ information.

	Stroke						Controls				
	Day from stroke to data collections (days)	Sex (M: male; F: female)	Age (years)	Walking speed (km/h)	Height (cm)	Weight (kg)	Sex (M: male; F: female)	Age (years)	Walking speed (km/h)	Height (cm)	Weight (kg)
Sub01	59	M	51	0.5	172	79	M	53	2	175	81
Sub02	49	M	51	1	173	59	M	51	2.2	170	60
Sub03	58	M	65	1.1	173	80	M	63	2	170	78
Sub04	54	F	84	1	156	50	F	84	2.4	157	53
Sub05	41	M	64	1.3	170	73	M	62	3.5	174	75
Sub06	55	M	81	0.6	167	59	M	79	1.1	165	60
Sub07	31	M	65	1.2	168	65	M	69	2.6	165	68
Sub08	48	M	77	0.6	164	62	M	76	1.5	167	65
Sub09	31	M	54	0.8	181	85	M	56	2.5	183	87
Sub10	73	F	26	2.5	165	51	F	25	2.4	166	49
Sub11	35	M	66	0.9	172	78	M	68	3.2	169	81
Sub12	85	F	70	0.2	157	59	F	67	3	160	61
Sub13	78	M	60	0.3	170	71	M	75	1.3	168	73
Sub14	34	M	77	0.5	177	82	M	74	1.4	176	83
Sub15	89	M	73	0.6	178	82	M	71	2.4	178	82
Sub16	30	M	43	0.8	177	80	M	41	2	175	78
Sub17	35	M	65	0.8	168	80	M	62	3.8	165	77
Sub18	32	M	61	0.6	181	81	M	62	1.4	177	79
Mean	50.9	15M/3F	62.9	0.85	170.5	71.7	15M/3F	63.2	2.26^*^	170	71.6
SD	19.5		14.3	0.51	7.2	11.5		14.3	0.77	6.6	11.2

^*^ indicated that there existed a signifcant difference between groups

### 3.2 Test of normality

The Shapiro–Wilk test revealed that *P*‐values of all outcomes of dependent variables were larger than 0.05, indicating that the data were normally distributed (Table 2).

**TABLE 2:** Test of normality (*P*‐value).

	ConD	ConND	StrI	StrP
*A*_0_	0.954	0.87	0.899	0.199
*A*_0_V	0.931	0.574	0.367	0.804
HarmNum	0.344	0.344	0.626	0.836
HarmNumV	0.48	0.598	0.752	0.135
MedianFre	0.09	0.305	0.993	0.069
MedianFreV	0.067	0.151	0.359	0.773
99.5% Fre	0.053	0.716	0.396	0.358
99.5% FreV	0.392	0.406	0.965	0.891
*H*_1_/*H*_2_ Ratio	0.661	0.268	0.108	0.349

Abbreviations: 99.5% Fre, 99.5% power frequency; 99.5% FreV, variability of 99.5% power frequency; *A*_0_V, variability of *A*_0_; ConD, controls’ dominant leg; ConND, controls’ non‐dominant leg; HarmNum, essential number of harmonics; HarmNumV, variability of essential number of harmonics; MedianFre, median power frequency; MedianFreV, variability of median power frequency; StrI, stroke survivors’ intact leg; StrP, stroke survivors’ paretic leg.

### 3.3 Interaction between the effect of stroke and the effect of different leg on each dependent variable

A significant interaction was observed in *A*_0_ (*F*_1, 34_ = 44.25, *P* < 0.001), number of essential harmonics (*F*_1, 34_ = 31.03, *P* < 0.001), *f*_median_ (*F*_1, 34_ = 21.64, *P* < 0.001) and *f*_99.5_ (*F*_1, 34_ = 52.36, *P* < 0.001). In short, regardless of healthy controls or stroke survivors, significant differences were found in *A*_0_, the number of essential harmonics, *f*_median_ and *f*_99.5_ compared to the two legs of healthy controls and the intact leg of stroke survivors. In short, *A*_0_, the number of essential harmonics and *f*_99.5_ were significantly smaller in the paretic leg of stroke survivors (*A*_0_: 61.71 ± 4.09; number of essential harmonics: 7.57 ± 1.12; *f*_99.5_: 4.19 ± 0.1) than the intact leg of stroke survivors (*A*_0_: 74.77 ± 6.65; number of essential harmonics: 10.87 ± 1.76; *f*_99.5_: 4.71 ± 0.31), the dominant leg of controls (*A*_0_: 80.05 ± 5.39; number of essential harmonics: 12.83 ± 3.18; *f*_99.5_: 4.46 ± 0.06) and the non‐dominant leg of controls (*A*_0_: 79.89 ± 4.09; number of essential harmonics: 12.50 ± 3.29; *f*_99.5_: 4.47 ± 0.1). *f*_median_ was observed to be significantly larger in the paretic leg of stroke survivors (*f*_median_ = 0.43 ± 0.03) than the intact leg of stroke survivors (*f*_median_ = 0.40 ± 0.01), the dominant leg in controls (*f*_median_ = 0.39 ± 0.01) and non‐dominant leg of controls (*f*_median_ = 0.39 ± 0.01). The *H*_1_/*H*_2_ ratio was significantly larger in the paretic leg of stroke survivors (*H*_1_/*H*_2_ ratio: 6.37 ± 1.44) than the intact leg of stroke survivors (*H*_1_/*H*_2_ ratio: 1.77 ± 0.23), the dominant leg in controls (*H*_1_/*H*_2_ ratio: 1.57 ± 0.09) and non‐dominant leg of controls (*H*_1_/*H*_2_ ratio: 1.56 ± 0.11). The detailed *post hoc* comparisons are shown in Figures 2, 3, 4, 5, 6, 7, Table 3.

**TABLE 3:** Statistical analysis for each dependent variables of vertical ground reaction force.

*A*_0_	ConD	StrI	ConND	StrP					
	80.05 (5.39)	74.77 (6.65)	79.89 (4.56)	61.71 (4.09)					
	**Groups × Legs**	**Legs**	**Groups**	**ConD vs. StrI**	**ConD vs. ConND**	**ConD vs. StrP**	**StrI vs. ConND**	**StrI vs. StrP**	**ConND vs. StrP**
	*P* < 0.0001	*P* < 0.0001	*P* < 0.0001	*P* = 0.137	*P* ≈ 1	*P* < 0.0001	*P* = 0.161	*P* < 0.0001	*P* < 0.0001
***A*_0_V**	**ConD**	**StrI**	**ConND**	**StrP**					
	2.52 (0.73)	3.05 (0.83)	2.48 (0.73)	3.77 (1.11)					
	**Groups × Legs**	**Legs**	**Groups**	**ConD vs. StrI**	**ConD vs. ConND**	**ConD vs. StrP**	**StrI vs. ConND**	**StrI vs. StrP**	**ConND vs. StrP**
	*P* = 0.022	*P* = 0.013	*P* = 0.001	*P* = 0.086	*P* ≈ 1	*P* < 0.0001	*P* = 0.043	*P* = 0.094	*P* < 0.0001
**HarmNum**	**ConD**	**StrI**	**ConND**	**StrP**					
	12.83 (3.18)	10.87 (1.76)	12.50 (3.29)	7.57 (1.12)					
	**Groups × Legs**	**Legs**	**Groups**	**ConD vs. StrI**	**ConD vs. ConND**	**ConD vs. StrP**	**StrI vs. ConND**	**StrI vs. StrP**	**ConND vs. StrP**
	*P* < 0.0001	*P* < 0.0001	*P* < 0.0001	*P* = 0.298	*P* ≈ 1	*P* < 0.0001	*P* = 0.693	*P* < 0.0001	*P* < 0.0001
**HarmNumV**	**ConD**	**StrI**	**ConND**	**StrP**					
	3.47 (0.73)	3.97 (0.65)	3.33 (0.75)	2.44 (0.83)					
	**Groups × Legs**	**Legs**	**Groups**	**ConD vs. StrI**	**ConD vs. ConND**	**ConD vs. StrP**	**StrI vs. ConND**	**StrI vs. StrP**	**ConND vs. StrP**
	*P* < 0.0001	*P* < 0.0001	*P* < 0.0001	*P* = 0.277	*P* ≈ 1	*P* = 0.003	*P* = 0.04	*P* < 0.0001	*P* = 0.031
**99.5% Fre**	**ConD**	**StrI**	**ConND**	**StrP**					
	4.46 (0.06)	4.71 (0.31)	4.47 (0.10)	4.19 (0.10)					
	**Groups × Legs**	**Legs**	**Groups**	**ConD vs. StrI**	**ConD vs. ConND**	**ConD vs. StrP**	**StrI vs. ConND**	**StrI vs. StrP**	**ConND vs. StrP**
	*P* < 0.0001	*P* < 0.0001	*P* < 0.0001	*P* = 0.035	*P* ≈ 1	*P* < 0.0001	*P* = 0.093	*P* < 0.0001	*P* < 0.0001
**99.5% FreV**	**ConD**	**StrI**	**ConND**	**StrP**					
	0.13 (0.05)	0.21 (0.06)	0.13 (0.03)	0.32 (0.11)					
	**Groups × Legs**	**Legs**	**Groups**	**ConD vs. StrI**	**ConD vs. ConND**	**ConD vs. StrP**	**StrI vs. ConND**	**StrI vs. StrP**	**ConND vs. StrP**
	*P* < 0.0001	*P* < 0.0001	*P* < 0.0001	*P* = 0.018	*P* ≈ 1	*P* < 0.0001	*P* = 0.002	*P* = 0.001	*P* < 0.0001
**MedianFre**	**ConD**	**StrI**	**ConND**	**StrP**					
	0.39 (0.01)	0.40 (0.01)	0.39 (0.01)	0.43 (0.03)					
	**Groups × Legs**	**Legs**	**Groups**	**ConD vs. StrI**	**ConD vs. ConND**	**ConD vs. StrP**	**StrI vs. ConND**	**StrI vs. StrP**	**ConND vs. StrP**
	*P* < 0.0001	*P* < 0.0001	*P* < 0.0001	*P* = 0.269	*P* ≈ 1	*P* < 0.0001	*P* = 0.043	*P* = 0.001	*P* < 0.0001
**MedianFreV**	**ConD**	**StrI**	**ConND**	**StrP**					
	0.005 (0.001)	0.007 (0.003)	0.005 (0.001)	0.015 (0.002)					
	**Groups × Legs**	**Legs**	**Groups**	**ConD vs. StrI**	**ConD vs. ConND**	**ConD vs. StrP**	**StrI vs. ConND**	**StrI vs. StrP**	**ConND vs. StrP**
	*P* < 0.0001	*P* < 0.0001	*P* < 0.0001	*P* = 0.031	*P* ≈ 1	*P* < 0.0001	*P* = 0.041	*P* < 0.0001	*P* < 0.0001
***H*_1_/*H*_2_ Ratio**	**ConD**	**StrI**	**ConND**	**StrP**					
	1.57 (0.09)	1.77 (0.23)	1.56 (0.11)	6.37 (1.44)					
	**Groups × Legs**	**Legs**	**Groups**	**ConD vs. StrI**	**ConD vs. ConND**	**ConD vs. StrP**	**StrI vs. ConND**	**StrI vs. StrP**	**ConND vs. StrP**
	*P* < 0.0001	*P* < 0.0001	*P* < 0.0001	*P* = 0.018	*P* ≈ 1	*P* < 0.0001	*P* = 0.017	*P* < 0.0001	*P* < 0.0001

99.5% Fre, 99.5% power frequency; 99.5% FreV, variability of 99.5% power frequency; *A*_0_V, variability of *A*_0_; BW, body weight; ConD, controls’ dominant leg; ConND, controls’ non‐dominant leg; HarmNum, essential number of harmonics; HarmNumV, variability of essential number of harmonics; MedianFre, median power frequency; MedianFreV, variability of median power frequency; StrI, stroke survivors’ intact leg; StrP, stroke survivors’ paretic leg.

### 3.4 Effect size

The partial eta squared values for interaction between the effect of stroke, and the effect of different legs were 0.565 for *A*_0_, 0.477 for essential number of harmonics, 0.389 for *f*_median_, and 0.606 for *f*_99.5_. These results indicated the large effect size (partial eta squared values >0.14).

## 4 DISCUSSION

Harmonic and power frequency analysis has been used to transform the vGRF from force–time curves into discrete parameters (*A*_0_, number of essential harmonics, *f*_median_ and *f*_99.5_). These parameters captured information about the entire waveform and can quantify vGRF patterns in ways that conventional local maximums and minimums cannot, such as identifying different vGRF pattern in stroke survivors during the early stages as shown in Figure 1. Therefore, applying frequency domain analyses should be preferred as a diagnostic tool for monitoring rehabilitation progress or assessing the severity of stroke survivors. The results in this present study were in line with hypotheses that *A*_0_, essential number of harmonics and *f*_99.5_ were significantly smaller in the paretic leg than the intact leg in stroke survivors and two legs in healthy controls. Unexpectedly, *f*_median_ was significantly greater in the paretic leg than the intact leg in stroke survivors and two legs in healthy controls.

### 4.1 The effect of stroke on *A*_0_

Indeed, vGRF patterns could be easily identified by visual inspection from Figure 1, but to the best of our knowledge, this was the first study using harmonic analysis to quantify the graphical differences in vGRF in stroke survivors. The key parameters for identifying differences in vGRF between control and stroke survivors were the magnitudes of the Fourier coefficients, as they significantly influence the shape of the force platform curves. Notably, *A*_0_ (percentage of BW), representing the mean value of the force signal throughout the stance phase, provided crucial information about the total impulse of the vGRF. This term was significantly lower in stroke survivors, indicating a reduced total vertical impulse in stroke gait compared to the control group and the intact leg. These findings aligned with previous observations from a review article (Hunnicutt & Gregory, 2017): paretic plantarflexor and knee extensor strength are significantly lower, muscle atrophy contributes to weakness, and neurological damage impairs muscle activation, leading to slower and asymmetric gait patterns. The paretic leg exhibited a significantly smaller *A*_0_ compared to the intact leg, influenced by both neurological damage and muscle atrophy. Similarly, our findings are consistent with the ranges established in previous literature (White et al., 2005), which utilized the same normalization convention and reported *A*_0_ values of 73.66% of BW for healthy controls (children, average age: 8.7 years old) and 60.02–66.75% of BW for patients with cerebral palsy (children, average age: 9.1 years old), indicating the sensitivity of this parameter to weight‐bearing deficits and confirming that significant reductions in *A*_0_ reflect the compromised vertical loading strategies inherent to neuromotor impairments. Addressing these deficits through harmonic coefficient was essential for improving motor function and gait in stroke survivors during rehabilitation. Another critical observation was that the significantly higher variability of the mean stance force (*A*_0_) observed in the paretic leg suggests a fundamental impairment in the consistency of weight‐bearing control. In stroke survivors, the damage to the corticospinal tract leads to a loss of fine motor regulation, forcing the central nervous system to rely on disinhibited, hyperexcitable brainstem pathways (e.g., reticulospinal and vestibulospinal tracts) for body support. This reliance on maladaptive subcortical networks likely introduces neural ‘noise’, manifesting as step‐to‐step fluctuations in vertical loading (*A*_0_). Consequently, this force variability reduces the predictability of the centre of mass trajectory. Since consistent weight acceptance is a prerequisite for safe forward progression, the inability to regulate *A*_0_ predictably may compromise dynamic stability, thereby contributing to the high prevalence of falls observed in this population (Li et al., 2018). Interestingly, there was no significant difference in the intact leg compared to healthy controls’ two legs. These observations suggested that these stroke survivors in the early stage primarily adjusted their paretic leg rather than the intact leg to generated appropriate impulses by stance‐to‐stance phases. This step‐by‐step adjustment required active control to maintain balance and stability during walking. Specifically, active control involved adjusting foot placement to stabilize the centre of mass to prevent falling. This was particularly important when proprioceptive feedback was compromised (the paretic leg, Bauby & Kuo, 2000).

### 4.2 The effect of stroke on essential number of harmonics of vGRF

When referring to a ‘lower essential number of harmonics’, it generally means that fewer harmonic components are necessary to accurately represent or analyse a signal, such as those used in gait analysis. From Figure 1, it is apparent that the waveforms in the paretic leg (inverse U shape) were easier to decompose into a simple sinusoidal wave compared to the waveforms in the intact leg (M shape). To elucidate the mathematical basis of the observed morphological changes, we applied a harmonic decomposition workflow (Figure 2) to the vGRF signals. This analysis revealed a fundamental simplification in the control of the paretic limb. In healthy controls and the intact limb, the vGRF profile is spectrally complex, characterized by a low harmonic amplitude ratio (*H*_1_/*H*_2_ ≈ 1.5); this indicates that a significant second harmonic (*H*_2_) is required to constructively interfere with the fundamental arch (*H*_1_) and generate the characteristic mid‐stance valley. Conversely, the paretic limb exhibited a drastic spectral reduction, with the ratio increasing approximately sixfold (*H*_1_/*H*_2_ ≈ 6). This dominance of the first harmonic confirms that the ‘inverse U’ profile is not merely a qualitative description but a quantifiable loss of the higher‐frequency force modulation (*H*_2_) necessary for dynamic weight acceptance and unloading, effectively reverting the gait strategy to a simplified, pendulum‐like oscillation. Therefore, a lower number of harmonics in the paretic leg suggested that the vGRF in the paretic leg is simpler, requiring fewer components to capture its essential features than the vGRF in the intact leg. This can also be explained by the fact that stroke survivors often face difficulties generating complex vGRF patterns. Consequently, the simpler shape of the vGRF in the paretic leg, compared to healthy controls, may help ensure gait stability and reduce the risk of falls. Additionally, it was possible that muscle strength in the paretic leg is insufficient to achieve complex motion, as discussed in the previous paragraph. When discussing the variability among all dependent variables, the variability in the essential number of harmonics was the only dependent variable that was lower in the paretic leg compared to both the intact leg and the controls' legs. This can be attributed to the fact that stroke survivors attempted to keep the shape of the vGRF in the paretic leg as consistent as possible to reduce the potential risk of tripping on the treadmill stance‐to‐stance phases.

### 4.3 The effect of stroke on *f*_99.5_ and *f*_median_ of vGRF

Firstly, it was essential to compare the values of *f*_99.5_ and *f*_median_ in the present study and previous studies to ensure the consistency of the calculation of the frequency domain. The values of *f*_99.5_ and *f*_median_ of controls in the present study were 4.47 Hz and 0.39 Hz compared to results of approximately 4.6 Hz and 0.36 Hz in McGrath et al.’s (2012) study and 4.54 Hz and 0.39 Hz in Wurdeman et al.’s (2011) study, indicating the consistency of these values among studies. The frequency domain content in the patients with stroke survivors in early stage had significant lower *f*_99.5_ but higher *f*_median_ in the paretic leg than intact leg in stroke survivors and both legs in healthy controls. Firstly, the lower *f*_99.5_ might be attributed to the slower walking speed in the paretic leg than in the intact leg (Hsiao et al., 2015). This hypothesis was supported by Wurdeman et al.’s (2011) and McGrath et al.’s (2012) studies that both patients with peripheral arterial disease and patients with multiple sclerosis walked significantly slower than heathy controls and led to the lower *f*_99.5_. For patients with multiple sclerosis, as a result of demyelination, nerve fibre function is compromised by the slowing of axonal conduction velocity (White & Dressendorfer, 2004). This slowing, combined with signal leakage, led to delayed muscle activation in the lower extremities. For patients with peripheral artery disease, previous research has identified reduced ankle plantarflexion power generation during late stance in both pain‐free and painful conditions compared to controls (Koutakis et al., 2010). This limitation may be linked to a restricted range of oscillatory phenomena within the neuromotor system in this plane of motion, which could contribute to the functional challenges associated with propulsion, leading to the slower walking speed and lower *f*_99.5_ than controls (McGrath et al., 2012; Wurdeman et al., 2011)_._ Similarly, lower *f*_99.5_ can be attributed to stroke survivors exhibiting abnormal muscle activation patterns due to the slower walking speed, including spasticity and reflex‐mediated coupling between different muscle groups (Li et al., 2018). Surprisingly, higher *f*_99.5_ in the intact leg of stroke survivors was observed compared to healthy controls. This could be the compensatory strategy. The intact leg typically moved faster and compensated for the weakness of the paretic leg by taking longer strides and generating more force (*A*_0_) during walking. Rather than representing a compensatory strategy, these observed differences likely serve as a direct quantification of the underlying motor impairment. The atypical vGRF harmonics and waveforms manifest as a mechanical consequence of the neurological insult, reflecting the paretic limb's inability to modulate force dynamically. While the intact leg exhibited longer strides and greater force generation (*A*_0_), this asymmetry is driven by the primary neuromuscular deficit of the paretic leg rather than an adaptive mechanism. This interpretation aligns with recent findings suggesting that such gait deviations stem from disrupted functional connectivity between motor areas, such as the supplementary motor area and premotor cortex (Peng et al., 2024), resulting in the observed mechanical constraints.

Unexpectedly, the higher *f*_median_ was observed in the paretic leg compared to the intact leg in stroke survivors and two legs in controls. This observation was opposite to the findings in patients with peripheral arterial disease and patients with multiple sclerosis, where lower *f*_median_ values were reported. In particular, McGrath et al. (2012) suggested that reduced *f*_median_ typically indicated more sluggish oscillatory components within the neuromotor system as the body applies force to the ground. The paradoxical observation of a higher *f*_median_ in the paretic leg, despite its morphologically simpler ‘inverse U’ profile, can be attributed to the spectral differences between sustained and transient loading strategies. As described by Li et al. (2018) the loss of corticospinal control in stroke survivors leads to a simplification of motor modules, replacing the complex, multi‐phase control of healthy gait with a merged extensor synergy. This results in a transient, arch‐like force profile (Figure 2). In contrast, the healthy ‘M’ profile represents a strategy of sustained weight‐bearing, geometrically approximating a flat‐topped plateau. In Fourier analysis, the zero‐frequency component (DC) represents the signal's mean magnitude; therefore, ‘plateau‐like’ waveforms possess a dominant DC component that concentrates the vast majority of the signal's power at 0 Hz. The paretic limb, lacking this sustained plateau, exhibits a relative reduction in this low‐frequency DC dominance. Consequently, without the massive concentration of energy at 0 Hz to ‘anchor’ the spectrum, the median power distribution naturally shifts upward, resulting in the higher *f*_median_ observed in the paretic leg.

### 4.4 Limitations

Several limitations regarding the measurement instrumentation must be acknowledged. First, the vGRFs were derived from the integration of capacitive pressure sensor data rather than measured directly by piezoelectric load cells. While this method provides a reliable estimation of vertical force, it may not be directly comparable to gold‐standard force plate measurements, particularly regarding shear forces which were not analysed here. Second, the sampling frequency of 100 Hz based on Wu et al.’s (2014) study, while sufficient for capturing the fundamental harmonics of gait (typically <20 Hz, Wu et al., 2014), is lower than the standard 1000 Hz often employed in force plate kinetics. This lower temporal resolution may attenuate higher‐frequency components or rapid transients at heel strike, potentially masking subtle harmonic differences. Additionally, data were collected on a motorized treadmill, which imposes a fixed walking speed and has been shown to alter gait kinetics and kinematics compared to overground walking. Consequently, the observed vGRF patterns should be interpreted within the context of treadmill‐based locomotion and may not fully generalize to natural, self‐selected overground gait. Finally, future studies may attempt to address the point in stroke progression where difference in GRF frequency become notable (3 months vs. 6 months of stroke survivors) and investigate the frequency domain in GRF in the medial–lateral and anterior–posterior direction using the gold standard instrumented treadmill.

### 4.5 Conclusion

This study demonstrates that harmonic and power spectrum analyses provide a robust, quantitative framework for characterizing neuromotor deficits in subacute stroke survivors. The analysis revealed that the paretic leg exhibits fundamental spectral simplification and instability distinct from healthy gait. Specifically, the significant reduction in mean stance force ($A_{0}$) and 99.5% power frequency (*f*_99.5_) quantifies the compromised weight‐bearing capacity and reduced neuromuscular output associated with slower walking speeds. Concurrently, the elevated variability of $A_{0}$ highlights a loss of step‐to‐step consistency, likely stemming from neural noise in compensatory subcortical pathways. Morphologically, the degradation of the vGRF from the healthy ‘M‐shape’ to the pathological ‘inverse U’ was mathematically captured by a lower essential number of harmonics and a drastic increase in the harmonic amplitude ratio (*H*_1_/*H*_2_ ratio). This elevated ratio reflects the suppression of the second harmonic (*H*_2_), which is essential for dynamic mid‐stance unloading, and a reversion to a primitive, fundamental loading pattern (*H*_1_). Finally, the paradoxical increase in median power frequency (*f*_median_) serves as a novel biomarker for this geometric change; it signals the loss of the sustained force plateau (and its dominant zero‐frequency DC component) required for stable single‐limb support.

Third, this geometric distortion paradoxically results in increased median power frequency (*f*_median_), a completely new biomarker of this mechanical phenomenon. Elevated *f*_median_ represents the loss of the braking and propulsion plateaus (and associated dominant DC or zero frequency‐component) necessary to sustain single limb support. Together, frequency‐domain measures provide greater sensitivity to these nuances than time‐domain kinematics. By assessing the loss of modulation specific to braking and propulsion periods, clinicians can objectively quantify impairment and baseline the return of dynamic stability in gait while personalizing rehabilitation for the subacute stroke population.

## AUTHOR CONTRIBUTIONS

Jung Hung Chien: Conceptualization, Formal analysis, Writing—original draft, Writing—review & editing, and Supervision. Shuisheng Fu: Conceptualization, Data curation, Writing—review & editing. Hongfang Yao: Conceptualization, Data curation, Writing—review & editing. Li Zhang: Conceptualization, Formal analysis, Data curation, Project administration, Writing—original draft, Writing—review & editing. All authors have read and approved the final version of this manuscript and agree to be accountable for all aspects of the work in ensuring that questions related to the accuracy or integrity of any part of the work are appropriately investigated and resolved. All persons designated as authors qualify for authorship, and all those who qualify for authorship are listed.

## CONFLICT OF INTEREST

All authors certify that they have no affiliations with or involvement in any organization or entity with any financial interest (such as honoraria; participation in speakers’ bureaus; membership, employment, consultancies, stock ownership, or other equity interest; and expert testimony or patent‐licensing arrangements), or non‐financial interest (such as personal or professional relationships, affiliations, knowledge or beliefs) in the subject matter or materials discussed in this manuscript.

## Data Availability

The datasets generated during and/or analysed during the current study are available from the corresponding author on reasonable request.
